# Putting menarche and girls into the global population health agenda

**DOI:** 10.1186/s12978-015-0009-8

**Published:** 2015-03-26

**Authors:** Marni Sommer, Carla Sutherland, Venkatraman Chandra-Mouli

**Affiliations:** Mailman School of Public Health, Columbia University, New York, USA; Independent Consultant, Cape Town, South Africa; World Health Organization, Geneva, Switzerland

**Keywords:** Menarche, Adolescent girls, Population health

## Abstract

Menarche, the onset of menstruation is a fundamental part of a girl’s transition from childhood to adolescence. Studies show that girls in many countries experience menarche with insufficient information and support. Girls from around the world report feeling ashamed and afraid. The potential health effects of such experiences include a weakening of girls’ sense of self-confidence and competence, which in turn may comprise girls’ abilities to assert themselves in different situations, including in relation to their sexuality and sexual and reproductive health. There is an important need for the public health community to assure that girls receive the education and support they need about menstruation, so they are able to feel more confident about their bodies, and navigate preventable health problems – now and in the future. For too long, the global health community has overlooked the window of opportunity presented by menarche. Family planning programs have generally focused their efforts on married couples and HIV programs have focused safer sex promotion on older adolescent girls and boys. Starting the conversation at menarche with girls in early adolescence would fully use this window of opportunity. It would engage young adolescent girls and be a natural first step for later, more comprehensive conversations about sexuality, reproduction and reproductive health. There are a number of initiatives beginning to tackle the provision of puberty information to girls and boys, but the global health community is overdue to set a global standard for the provision of such guidance.

Menarche, the onset of menstruation and part of a girl’s transition from childhood to adolescence, is a critically important but under-recognized public health issue. Evidence from many countries indicates that many girls start their menstruation uninformed, unprepared, and unsupported to manage their monthly menstrual periods [1-3]. Girls report hiding the onset of menses from others and missing school due to fear of a shameful menstrual leak. They demonstrate a lack of understanding of why menstruation occurs, how it relates to fertility, and when to expect their monthly periods [4-8]. At the start of this normal biological phenomenon of sexual maturation, girls around the world report feeling afraid, ashamed, and confused [4,9]. Some of these challenges may arise from cultural taboos around menstruation, from adult silences around discussing sexual maturation, or from misinformation provided to them from a variety of sources (e.g. peers, parents, teachers). The result is that girls begin their periods without fully understanding what is happening to their bodies.

What are the health effects of these experiences? Starting menstruation in ignorance and in fear may weaken girls’ sense of self-confidence and competence [10-12]. These deficits could also compromise girls’ future abilities to assert for themselves in situations regarding their sexuality and to maintain their sexual and reproductive health [13,14]. Existing evidence from high-income countries suggests girls reaching menarche and puberty early, without adequate emotional support, are likely to engage in earlier sexual relations and substance abuse, posing risks for adolescent pregnancy and other negative health outcomes [15-17].

Educating girls about menstruation, what it means, and how it can be safely and healthily navigated, and providing them with the physical and emotional support needed to manage their monthly menstruation with confidence, will enable girls to take greater charge of their lives, feel more positive about themselves and their bodies, and may help to mitigate subsequent preventable health problems.

For decades the global public health community has devoted considerable resources—appropriately so— to strengthening family planning and maternal health education and programs. Remarkably, however, these efforts have failed to directly and explicitly address menarche. National family planning programs in low and middle-income countries have generally focused their efforts on married women. Given that young married women are often under heavy pressure to bear children, they represent only a small portion of family planning clients. In addition, due to legal and social barriers to access, young unmarried adolescents often fail to seek contraception, nor are they targeted for the provision of contraception through public health programs focused on family planning or maternal health. This gap represents a missed opportunity. The onset of menstruation could provide a natural entry point for beginning to educate a girl about her reproductive capacity and contraceptive choices.

In response to the HIV epidemic, the global public health community actively promoted sexuality education. In countries where these programs are in place (and there are many countries where they are not), they focus on promoting safer sex among older adolescents. There has continued to be enormous resistance from parents, teachers, religious leaders and community members to the provision of sexuality education for young people, and even more so for very young adolescents (ages 10–14). Such programs have also frequently overlooked menarche. Puberty education – and education on menstruation in particular – is likely more culturally and politically acceptable than education on safer sex. It may also provide a useful first step for parents, schools, and other public health programming towards the provision of education on sexuality and contraception.

What should be done? Prior to menarche, and the potentially frightening experience of seeing blood for the first time, every girl should be informed about what menstruation is, why it occurs, and how to deal with it. Every girl should also be given practical support (e.g. sanitary menstrual materials and washing facilities) and emotional guidance to handle her monthly periods. Initiatives in several countries have begun to address these needs. Research in Iran and India has demonstrated the beneficial effects of educating girls about menstruation and menstrual hygiene [18,19]. A four-year initiative by Columbia University has launched locally designed puberty booklets for girls and for boys in Tanzania, Ghana, Ethiopia, and Cambodia. These have been embraced by the Ministries of Education in all four countries [20]. Similar initiatives have been launched by Save the Children in Nepal and Malawi, and by Georgetown University in Rwanda and Guatemala. UNESCO recently launched a major new initiative to improve teachers’ abilities to educate and support girls in classrooms [21]; and Proctor & Gamble, a major producer of sanitary products globally, launched a puberty education program for girls [22].

Non-governmental organizations such as Strategies for Hope and the Families Matter Program are educating and encouraging parents to communicate with their daughters about puberty and menstruation in a number of countries [23,24]. UNICEF has integrated menstruation education with its efforts to improve water, sanitation and disposal facilities in schools [4]. The benefits of providing low cost sanitary products and puberty education have been demonstrated in Ghana [25]. The World Health Organization is working to improve the competencies of health care workers to provide effective and empathic care to girls with menstrual health problems [26]. Population Council has developed the iMatter curriculum, developed for 5^th^ and 6^th^ graders (10–12 year olds) in the U.S., which lays the groundwork for adolescent sexual health through puberty education and social and emotional learning [27]. Girls and boys have welcomed and benefited from these initiatives. Equally important, parents, heads of schools and teachers, religious leaders, and health workers have enthusiastically supported these efforts.

While these initiatives are important and promising first steps, they are not enough. Far too many girls across the low-income world are struggling with almost complete ignorance of their normal biological maturation and its consequences. It is time to move from pioneering efforts to a global standard of what girls and women deserve.
